# The long‐term outcome of neuropsychological function is favorable in patients with non‐malignancy related anti-GABA_B_R encephalitis: a case series

**DOI:** 10.1186/s12883-021-02111-0

**Published:** 2021-02-23

**Authors:** Caihong Ji, Dengchang Wu, Zhongqin Chen, Kang Wang

**Affiliations:** Department of Neurology, The First Affiliated Hospital, Zhejiang University School of Medicine, Hangzhou, China

**Keywords:** Anti-GABA_B_R encephalitis, Non‐malignancy, Neuropsychological function, Long‐term outcome, Adult patients

## Abstract

**Background:**

Anti-GABA_B_R encephalitis is a rare type of autoimmune encephalitis, which often presents with memory impairments, behavioral changes and seizures. This case series describes the neuropsychological function recovery pattern in five adult patients with anti-GABA_B_R encephalitis.

**Case presentation:**

We recruited five patients with clinically confirmed anti-GABA_B_R encephalitis without any accompanying malignancy. Comprehensive neuropsychological evaluation was conducted on each patient. All the five patients were evaluated in the chronic phase. Five age and gender matched healthy adults were recruited as control group. Our study demonstrated that the neuropsychological function of the patients with anti-GABA_B_R encephalitis was no different with respect to the control group during the chronic phase (more than 6 months after onset). Moreover, one patients with neuropsychological evaluation at acute (within 2 months after onset of symptoms), post-acute (2 to 6 months after onset) and chronic phases respectively, presented neuropsychological function recovered as early as in the post-acute phase and only showed cognition impairment in the acute phase.

**Conclusions:**

The results of this retrospective study indicate a favorable long-term neuropsychological function outcome in adult patients with anti-GABA_B_R encephalitis, despite severe memory deficits occurring during the acute phase. These findings improve our understanding related to the prognosis of neuropsychological function in anti-GABA_B_R encephalitis.

## Background

Autoimmune encephalitis encompass a group of rare conditions which lead to inflammation of the central nervous system and they occur as a result of antibody reactions to the neuronal surface and synaptic proteins [1]. Several autoantibodies have been identified, including those against the gamma-aminobutyric acid-B (GABA_B_) receptor, N-methyl-D-aspartate (NMDA) receptor, α-amino-3-hydroxy-5-methyl-4-isoxazolepropionic acid (AMPA) receptor et al. [2].

GABA is an inhibitory neurotransmitter and decreases nerve cell activity by binding to GABA_A_ and GABA_B_ receptors [3]. Lancaster et al. first reported 15 patients who were positive for anti-GABA_B_R antibodies in cerebrospinal fluid (CSF), and their clinical symptoms included refractory epilepsy, memory loss, abnormal behavior, and damaged consciousness [1]. Given that anti-GABA_B_R encephalitis mainly involves the limbic system, not surprisingly, memory deficits were a presenting symptom in these patients [4, 5]. Kim et al. reported five patients with anti-GABA_B_R encephalitis, and most of them presented with memory deficits [2]. Besides memory impairments, anti-NMDAR encephalitis patients also presented with deficits in executive function [6]. However, there was no comprehensive neuropsychological assessments in those anti-GABA_B_R encephalitis patients reported so far. Additionally, most of these patients were found to have a concomitant neoplasm, making the long-term follow-up of neuropsychological function more difficult [7, 8].

In light of this, in the current study, we retrospectively investigated the neuropsychological function impairments in the acute, post-acute, and chronic phase, respectively, in adult patients with anti-GABA_B_R encephalitis. This study sought to provide insights into the recovery pattern of neuropsychological function in anti-GABA_B_R encephalitis patients and its lone-term outcome. This, would help to uncover the mechanism of neuropsychological function impairment in these patients.

## Case presentation

Five adults with CSF antibody-confirmed anti-GABA_B_R encephalitis were selected from those patients admitted to our hospital, the First Affiliated Hospital, Zhejiang University School of Medicine, during the period from October 2014 to January 2018. We included all the anti-GABA_B_R encephalitis patients in our hospital during that period. The diagnosis was based on typical clinical symptoms, including confusion, decreased cognitive function and seizures. All the five patients had seizures and three of the patients had behavioral and psychological symptoms, like drowsiness and aggressive behavior in the acute phase. Furthermore, and most importantly, anti-GABA_B_ receptor antibodies were present positive in the CSF (2). Malignancy was excluded by extensive whole-body workup. The mean age was 50.20 ± 10.08 years and the included patients were three females and two males. Cell-based indirect immunofluorescence (IIF) tests were used to detect autoantibodies in both serum and CSF. Testing items included anti-NMDA receptor, anti-GABA_B_ receptor, anti-AMPA receptor, anti-leucinrich glioma-inactivated 1 (LGI1), and anti–contactin-associated protein-like 2 (Caspr2). All the patients were negative for other antibodies while positive for anti-GABA_B_ receptor antibody. All the subjects enrolled had MRI scans. One patient presented a low signal for the T1-weighted image and a high signal intensity for the T2-weighted image in the bilateral medial temporal lobe, while the other four patients presented normal MRI. All the patients did long-term video electroencephalogram (EEG) monitoring. Some patients had abnormal EEG results. For example, Patient 1 had left temporal spikes and a small amount of 1-3Hz slow waves in the bilateral frontal lobe. Patient 3 showed left temporal spikes. She also had a seizure attack during EEG monitoring which presented with absence, and the synchronous EEG displayed rhythmic theta activity (6 Hz) in the left temporal electrode chain (maximal at T3-T5) for 20 seconds, which then returned to baseline. Patient 4 still had left temporal spikes and subclinical seizure attack in the chronic phase.

All the patients were treated with intravenous immunogloblin (IVIG; 0.4 g/kg/day for five days). Three of the patients were treated with methylprednisolone 500 mg for three days and the doses were reduced gradually. One of the patients was treated with dexamethasone 10 mg. Three of the patients were also treated with immunosuppressive therapy, cyclophosphamide or azathioprine. All the patients recovered well after treatment with the modified Rankin Scale (mRS) scores of 0–1 in the chronic phase.

Five healthy adults were enrolled as healthy controls and they did not report any previous history of neurological disorders. All of them were individually matched to the anti-GABA_B_R encephalitis patients in terms of gender, degree of education, and age. The Ethics Committee of the First Affiliated Hospital, Zhejiang University School of Medicine approved the study. Written informed consent was obtained from each participant.

All the participants completed the same neuropsychological scales. The whole work was conducted entirely by Dr. CJ, who was specifically trained in neuropsychological assessment. The tests assessed a number of neuropsychological fields, (1) Executive control: Stroop test (both color dot and word tests); (2) Information processing speed: symbol-digit modalities test (SDMT); (3) Working memory: digit span test (both forward and backward); (4) Semantic fluency test: fruit and vegetables, animals; (5) Visual-spatial capacity: block design test; (6) Verbal memory test (Chinese version of the Verbal Learning Test, CAVLT), and nonverbal memory test (Aggie Figures Learning Test, AFLT) [9]. All of the patients finished neuropsychological function assessment in the chronic period (6–36 months after disease onset) when all the treatment had been terminated and patients had returned back to work. And several patients were also evaluated at the acute phase (within 2 months after onset of symptoms) and post-acute (2 to 6 months after onset). The Kolmogorov–Smirnov test was used to investigate the distribution of each variable. The quantitative variables between groups were compared using the independent t-test, or Mann–Whitney test, according to the distribution of the samples.

Both patients and controls’ demographic and clinical information are presented in Table 1. No significant difference was observed between the patients and controls in terms of gender, age, or the level of education (Table 2). All of the patients’ clinical symptoms were remarkably relieved following cessation of the treatment. All of them were seizure free in the chronic phase. None of the patients developed a neoplasm during the period of long-term follow up, which ranged from 1.5 to 5 years.

**Table 1:** Clinical and demographic information of the patients and healthy controls

	Sex	Age	Education	Acute disease symptoms	Neoplasms	MRI	Treatment	Duration of disease when did neuropsychological tests
Patient_1 (HLP)	F	35	12	seizures	none	abnormal	IVIg, Steroids, AEDs	1 m, 4 m, 2.5y
Patient_2	M	49	5	seizures, behavioral and psychological symptoms	none	normal	IVIg, Steroids, AEDs	1 m, 1.5y
Patient_3	F	42	16	seizures	none	normal	IVIg, Steroids, AEDs	5 m, 2y
Patient_4	M	59	3	seizures, behavioral and psychological symptoms	none	normal	IVIg, Steroids, AEDs	3y
Patient_5	F	61	4	seizures, behavioral and psychological symptoms	none	normal	IVIg, AEDs	1y
Control_1	F	35	7					
Control_2	M	49	9					
Control_3	F	42	5					
Control_4	M	60	8					
Control_5	F	67	15					

*AEDs *antiepileptic drugs, *IVIg *intravenous human immunoglobulin

**Table 2:** Demographic characteristics of the patients and healthy controls

	Patients (Mean ± SD)	HCs (Mean ± SD)	t	*p*
Age (years)	50.20 ± 10.08	50.60 ± 13.01	-0.054	0.958
Education (years)	8.20 ± 5.59	8.80 ± 3.77	-0.199	0.847
Gender	3 females; 2 males	3 females; 2 males		

As shown in Table 3, no significant differences between patients and controls were found in the Stroop test (color dot or color word), SDMT, digit span test (forward or backward), semantic fluency test (fruit and vegetables or animal), block design test, CAVLT (immediate memory following interference, delayed recall, recognition), or AFLT (immediate memory following interference, delayed recall, recognition) in the chronic period.

**Table 3:** The results of neuropsychological tests of the patients and healthy controls in the chronic phase

Tests	Patients (*n* = 5)	Controls (*n* = 5)	*p*
Stroop test (color dot)	22.50 ± 8.65	18.51 ± 2.42	0.35
Stroop test (color word)	37.81 ± 11.78	35.77 ± 7.56	0.75
SDMT	33.80 ± 15.27	30.80 ± 8.07	0.71
Digit span test (forward)	6.80 ± 1.10	8.20 ± 1.79	0.19#
Digit span test (backward)	4.60 ± 2.07	4.00 ± 1.73	0.50
Semantic fluency test (vegetable and fruit)	16.00 ± 7.52	19.40 ± 6.07	0.45
Semantic fluency test (animal)	12.80 ± 4.32	16.00 ± 3.46	0.23
Block design test	27.80 ± 11.88	25.20 ± 6.26	0.91#
CAVLT (immediate memory)	7.20 ± 3.96	9.00 ± 3.00	0.44
CAVLT (delayed recall)	5.00 ± 5.00	8.80 ± 2.17	0.16
CAVLT (recognition)	10.60 ± 4.51	14.20 ± 1.30	0.13#
AFLT (immediate memory)	7.00 ± 5.10	7.20 ± 2.39	0.94
AFLT (delayed recall)	6.80 ± 5.36	7.00 ± 3.81	0.95
AFLT (recognition)	10.80 ± 4.76	13.40 ± 1.67	0.45#

*SDMT *symbol-digit modalities test, *CAVLT *Chinese auditory verbal learning test, *AFLT *Aggie Figures Learning Test

# Mann–Whitney test

## Case presentation

HLP was a 34 year-old woman without previous psychiatric history. She suffered from repeated seizures for half a month prior to admission at our hospital. During her admission, brain MRI revealed a high signal in the bilateral medial temporal lobe. Long-term EEG monitoring showed left temporal spikes. The HLP’s CSF panel displayed positivity for the anti-GABA_B_R antibodies (1:32) and the serum titer of anti-GABA_B_R antibodies was 1:320. HLP was treated by IV steroids, IVIG, and IV cyclophosphamide. She also underwent AEDs therapy. Repeat anti-GABA_B_R antibodies titer in CSF was normal (less than 1:10) at 3 months after onset of the disease.

**Fig. 1:**
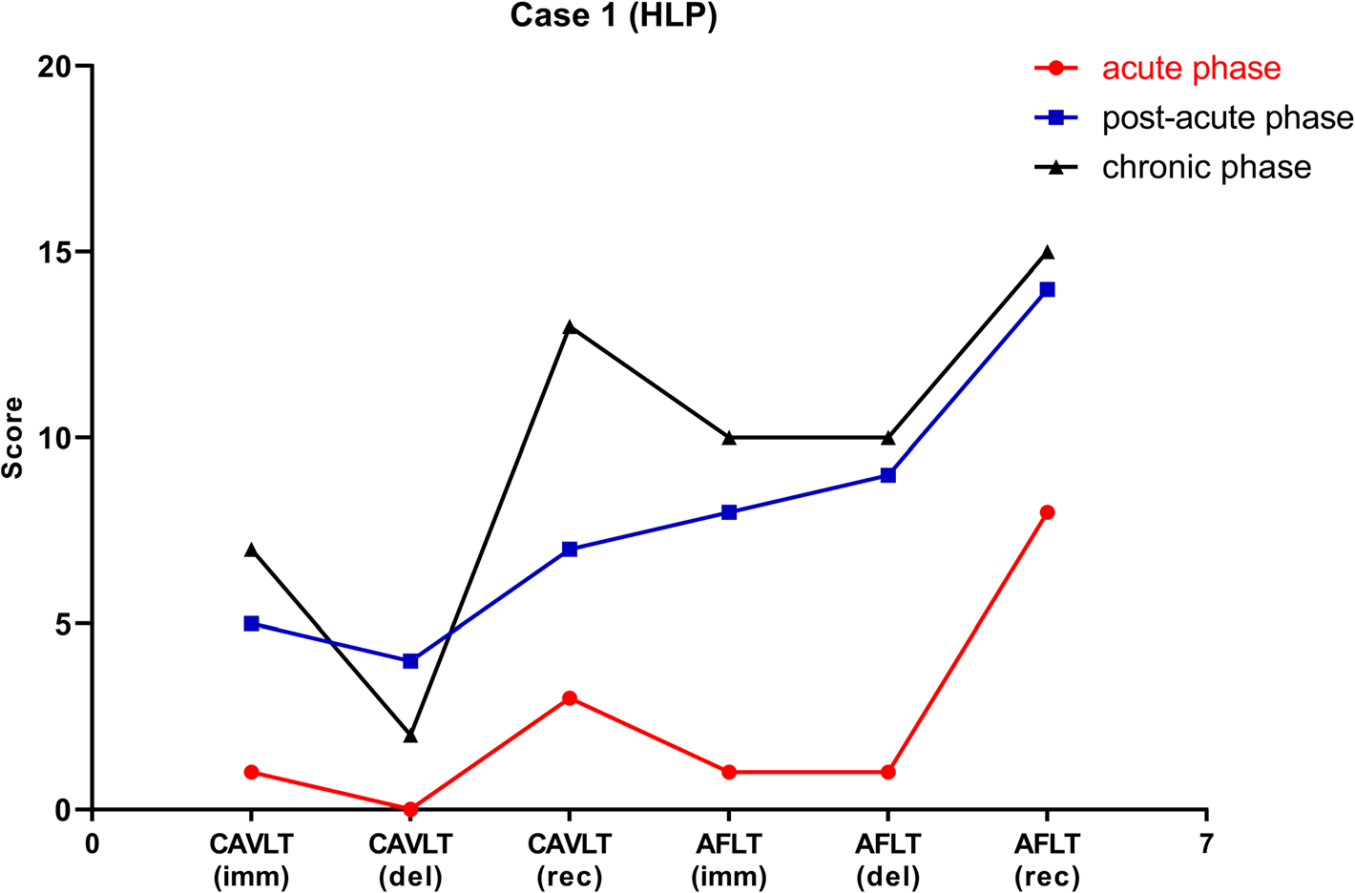
The results of neuropsychological function in one patients with non-malignancy related anti-GABA_B_R encephalitis. The patient’s verbal memory (CAVLT) and nonverbal memory (AFLT) were severely damaged in the acute phase, but recovered dramatically in the post-acute phase, which were similar with the scores in the chronic phase. Red line, acute phase; Blue line, post-acute phase; Black line, chronic phase. AFLT, Aggie Figures Learning Test; CAVLT, Chinese version of the Verbal Learning Test; imm, immediate memory following interference; del, delayed recall; rec, recognition

HLP performed the cognitive tests at one month, four months and 2.5 years after disease onset, respectively (Fig. 1). Results of cognitive testing suggested that her memory was severely damaged in the acute phase. During the acute phase, she could only recall 1, 0, and 3 words in immediate recall, delayed recall, and recognition, respectively in CAVLT, while for AFLT, it was 1, 1, and 8 words in immediate recall, delayed recall, and recognition, respectively. In the post-acute phase, her memory function was much improved. She could recall 5, 4, and 7 words in immediate recall, delayed recall, and recognition, respectively in CAVLT, while 8, 9, and 14 words in AFLT. The results of CAVLT and AFLT, in the chronic phase, were similar to those observed in the post-chronic phase.

## Discussion and conclusions

Our retrospective study demonstrated that the neuropsychological function of patients with non-malignancy related anti-GABA_B_R encephalitis had almost recovered in the chronic phase. Moreover, one patients with neuropsychological evaluation at acute, post-acute and chronic phases respectively, presented neuropsychological function recovered as early as in the post-acute phase and only showed cognition impairment in the acute phase.

Since the affected site in anti-GABA_B_R encephalitis is mainly in the hippocampus [2], the clinical feature of these patients is represented by memory function impairment, both in verbal and nonverbal memory, in the acute phase. Previous animal studies displayed that, without GABA_B_ receptors, the mice developed problems in terms of memory, learning, and behavior, symptoms similar to those observed in anti-GABA_B_R encephalitis, thus suggesting dysfunction of the limbic system [10, 11].

In this study, we first reported the long-term neuropsychological function outcome in patients with non-malignancy related anti-GABA_B_R encephalitis. The patients showed no impairment in all the neuropsychological function domains in the chronic phase. In turn, we conjecture that there could be two reasons for this favorable outcome in neuropsychological function in our patients. Firstly, all the five patients did not present with an neuroendocrine tumor of the lung or small cell lung cancer, which are common in anti-GABA_B_R encephalitis [8]. We followed up all the five patients for a long period, ranging from 1.5 to 5 years, and no tumor was discovered. Previous studies also reported that the mRS (modified Rankin Scores) were between 0 and 1 in anti-GABA_B_R encephalitis cases without a tumor in the follow-up period, which ranged from 3 to 20 months, and these were much better than those with cancer [8]. Secondly, previous studies have concluded that early immunotherapy was a favorable indicator of neuropsychological function outcome in anti-NMDAR encephalitis [6]. However, no similar studies have been conducted in anti-GABA_B_R encephalitis. Given that anti-NMDAR and anti-GABA_B_R encephalitis are both autoimmune encephalitis, we hypothesize that the pathophysiological mechanisms of the two disease are similar. All the patients included in this study received immunotherapy during the early stages of the disease (within two months after onset of the first symptom), which may have contributed to the favorable outcomes in terms of neuropsychological function.

The current study also found that the recovery pattern of neuropsychological function in anti-GABA_B_R encephalitis was similar to anti-NMDAR encephalitis, in accordance with our previous study [12], and that the patient made a nearly full cognitive recovery as early as 4 months following onset of the symptoms. Our previous study also assessed neuropsychological function in five patients with anti-NMDAR encephalitis at 1–2, 6, and 11–12 months after treatment, respectively. And we found the anti-NMDAR encephalitis group showed no significant differences in verbal memory compared with the control group 6 months after the immunotherapy [12]. Here, we also found that in anti-GABA_B_R encephalitis there was no remarkable difference between post-acute and chronic phases in neuropsychological tests. It seems that the cognitive recovery reached a plateau within 6 months following disease onset. This finding may encourage these patients to return to routine work and study as early as possible since their cognition function will have recovered.

The results of the retrospective study indicated favorable long-term neuropsychological function outcome in adult patients with anti-GABA_B_R encephalitis, despite severe memory deficits in the acute phase. Our patient also presented neuropsychological function recovered as early as in the post-acute phase. These findings improved our understanding of the neuropsychological function prognosis in anti-GABA_B_R encephalitis.

## Data Availability

All data used and/or analyzed during the study is available on request from the corresponding author.

## Abbreviations

AFLT: Aggie Figures Learning Test
AMPA: α-amino-3-hydroxy-5-methyl-4-isoxazolepropionic acid
Caspr2: Contactin-associated protein-like 2
CAVLT: Chinese version of the Verbal Learning Test
CSF: Cerebrospinal fluid
GABA_B_R: Gamma-aminobutyric acid-B receptor
IVIG: Intravenous immunogloblin
LGI1: Leucinrich glioma-inactivated 1
NMDA: N-methyl-D-aspartate
SDMT: Symbol-digit modalities test
VEEG: Video electroencephalogram
